# *UBE2C* Is a Transcriptional Target of the Cell Cycle Regulator FOXM1

**DOI:** 10.3390/genes9040188

**Published:** 2018-03-29

**Authors:** Pedro Nicolau-Neto, Antonio Palumbo, Marco De Martino, Francesco Esposito, Tatiana de Almeida Simão, Alfredo Fusco, Luiz Eurico Nasciutti, Nathalia Meireles Da Costa, Luis Felipe Ribeiro Pinto

**Affiliations:** 1 Programa de Carcinogênese Molecular, Instituto Nacional de Câncer—INCA, Rua Andre Cavalcanti 37, Rio de Janeiro 20231-050, RJ, Brazil; pedronicolau.n@gmail.com (P.N.N.); natmeireles@gmail.com (N.M.D.C.); 2 Laboratório de Interações Celulares, Instituto de Ciências Biomédicas, Universidade Federal do Rio de Janeiro, Prédio de Ciências da Saúde—Ilha do Fundão, A. Carlos Chagas, Rio de Janeiro 21941-902, RJ, Brazil; palumbo@icb.ufrj.br (A.P.J.); luiz.nasciutti@isto.ufrj.br (L.E.N.); 3 Istituto di Endocrinologia e Oncologia Sperimentale—CNR c/o Dipartimento di Medicina Molecolare e Biotecnologie Mediche, Università degli Studi di Napoli “Federico II”, 80131 Naples, Italy; marco.demartino2@unina.it (M.D.M.); francesco.esposio2@unina.it (F.E.); alfusco@unina.it (A.F.); 4 Departamento de Bioquímica, Instituto de Biologia Roberto Alcantara Gomes, Universidade do Estado do Rio de Janeiro, Av. 28 de Setembro 87, Fundos, Pavilhão Américo Piquet Carneiro—4° Andar, Rio de Janeiro 20551-030, RJ, Brazil; tasimao@gmail.com

**Keywords:** FOXM1, UBE2C, Gene expression control, Cancer

## Abstract

FOXM1 (forkhead box protein M1) is a transcription factor that participates in all stages of tumor development, mainly through the control of cell cycle and proliferation, regulating the expression of genes involved in G1/S and G2/M transition and M phase progression. The ubiquitin conjugating enzyme E2 (UBE2C) is a member of the anaphase promoting complex/cyclosome, promoting the degradation of several target proteins along cell cycle progression, during metaphase/anaphase transition. FOXM1 and UBE2C have been found overexpressed in a wide range of different solid tumors. Therefore, the aim of this study was to investigate whether *UBE2C* is a transcriptional target of FOXM1, using esophageal squamous cell carcinoma (ESCC) as a model, in addition to several cancer-deposited data. Our results show that *FOXM1* and *UBE2C* expression present a positive correlation in normal tissues and in 25 distinct tumor types, including ESCC, where these genes are overexpressed. Moreover, FOXM1 binds to *UBE2C* promoter region in ESCC cell line and transcriptionally activates it, leading to UBE2C upregulation. In conclusion, this study provides evidences that FOXM1 transcriptionally regulates *UBE2C* expression in ESCC and their deregulation may be a general phenomenon in human neoplasias.

## 1. Introduction

Forkhead box protein M1 (FOXM1) is one of the most studied members of the FOX transcriptional regulators family [1], characterized by an evolutionary conserved ‘forkhead’ or ‘winged-helix’ DNA-binding domain (DBD) [2]. FOXM1 is overexpressed in a wide range of different solid tumors [3,4], including esophageal squamous cell carcinomas (ESCC) [5,6]. FOXM1 functions as an oncogene, contributing, among other processes, to the loss of control of cell cycle progression and cell proliferation, since this protein is a key regulator for G1/S and G2/M transition and M phase progression. The main mechanism by which FOXM1 controls these cellular features is through the transcriptional regulation of key genes involved in cell cycle control [7].

As a member of the anaphase promoting complex/cyclosome (APC/C), ubiquitin conjugating enzyme E2 (UBE2C) promotes the degradation of several target proteins along cell cycle progression, particularly mitotic cyclins, during metaphase/anaphase transition [8,9]. We have recently shown that UBE2C is upregulated in ESCC samples, and its abrogation altered the proliferation and cell cycle profile of ESCC cell lines, by directly modulating cyclin B1 expression levels, demonstrating the participation of UBE2C in critical steps of ESCC genesis [10]. Recently, a positive correlation between *UBE2C* and *FOXM1* gene expression in esophageal adenocarcinoma (EAC) samples and in vitro EAC-derived cells was reported [11].

In this way, the aim of this study was to investigate whether *UBE2C* is a transcriptional target of FOXM1, using ESCC as a model. This study shows that *FOXM1* and *UBE2C* are overexpressed and positively correlated in ESCC as well as in a wide range of distinct tumor types. In silico analysis showed that FOXM1 binds to *UBE2C* promoter in different tumors. Finally, FOXM1 binds to its response elements within *UBE2C* promoter, transcriptionally activates it and leads to increased levels of UBE2C protein in ESCC cell line, demonstrating that *UBE2C* is a FOXM1 transcriptional target.

## 2. Materials and Methods

### 2.1. Patients and Samples

Gene expression analysis comprised 52 paired ESCC and non-malignant histologically normal surrounding mucosa (collected at least 4 cm far from the tumor border) biopsy samples from patients who were submitted to endoscopy, from 2006 to 2013 at the Brazilian National Cancer Institute (INCA). None of the patients comprised in this study had undergone any type of chemotherapy and/or radiotherapy. At the moment of the endoscopy, clinicopathological data were obtained, by using a standardized questionnaire. The clinicopathological characteristics of the ESCC patients enrolled in this study are described in Supplementary Materials Table S1.

### 2.2. Ethics

Written informed consent was obtained from all the study participants. The project was approved by the Ethics Committee of the Brazilian National Cancer Institute (number of the project: 116/11). All methods were performed in accordance with the relevant guidelines and regulations.

### 2.3. RNA Extraction, Reverse Transcription and qRT-PCR

Total RNA was extracted from tissue samples using RNEasy mini kit (Qiagen, Hilden, Alemanha), following the manufacturer’s protocol. RNA samples yields were measured using NanoDrop (Thermo, Waltham, MA, USA) and 500 ng of total RNA was reverse transcribed using SuperScript II (Invitrogen, Carlsbad, CA, USA), according to the manufacturer’s protocol. *FOXM1* and *UBE2C* expression levels were assessed by real-time PCR, using a rotor-gene platform (Qiagen). Specific oligonucleotides were used in the expression levels analyses, as follows: *FOXM1* Forward: 5′ AACCTTTCCCTGCACGACAT 3′, *FOXM1* Reverse: 5′ GGTCCAGTGGCTTAAACACC 3′, *UBE2C* Forward: 5′ TGGTCTGCCCTGTATGATGT 3′, *UBE2C* Reverse: 5′ AAAAGCTGTGGGGTTTTTCC 3′; *GAPDH* Forward: 5′ CAACAGCCTCAAGATCATCAGCAA 3′, *GAPDH* Reverse: 5′ AGTGATGGCATGGACTGTGGTCAT 3′. Each reaction consisted of 5.0 μL of Quantifast SYBR Green PCR Master Mix (Qiagen), 10 pmols of primers and 1 μL of cDNA. The amplification reaction was performed as follows: 5 min for DNA pre-denaturation at 95 °C, followed by 40 cycles of hybridization and complementary chain synthesis for 5 s at 95 °C and 10 s at 60 °C.

### 2.4. Cell Line and Transfections

The ESCC-derived cell line, TE-1, was kindly donated by Dr. Pierre Hainaut (University of Grenoble, France). TE-1 cells were cultured in RPMI medium (Invitrogen) supplemented with 10% fetal bovine serum (Thermo) and 1% of the cocktail penicillin/glutamine/streptomycin (Invitrogen) and maintained at 37 °C under 10% CO_2_. TE-1 cells had FOXM1 expression levels induced by transfecting different amounts of an expression vector (pcDNA3-FOXM1) [12] or the empty backbone vector (pcDNA3, Invitrogen), used as an experimental control, using Lipofectamine 2000 (Invitrogen), following the manufacturer’s protocol. Cells were authenticated using Powerplex 16 STR System (Promega, Madison, WI, USA) and were routinely tested for mycoplasma using a Mycosensor (Agilent, Santa Clara, CA, USA).

### 2.5. Chromatin Immunoprecipitation (ChIP) Assay

TE-1 cells (2 × 10^6^ cells/plate/10 mL) were plated in 10 mm plates, transfected with a FOXM1 expression vector (total of 5 µg of pcDNA3-FOXM1 or empty pcDNA3/plate/10 mL) and, 24 h later, 1% formaldehyde was added to the culture medium for 10 min at room temperature to cross-link proteins to DNA and then neutralized by the addition of 125 mM glycine pH 2.5. Cells lysis, immunoprecipitation and DNA extraction were performed using the SimpleChIP^®^ Enzymatic Chromation IP Kit (Magnetic Beads), according to the manufacturer’s protocol (Cell Signaling, Danvers, MA, USA). Lysates were sonicated, according the following: 12× (5 s on: 5 s off) at 21% amplitude, using the sonicator Vibra cell 75041. FOXM1 immunoprecipitation was performed using 9 μg of FOXM1 antibody (FOXM1 (C-20): sc-502, Santa Cruz, Dallas, TX, USA). Immunopreciptated DNA was amplified by qPCR, using primers for FOXM1-binding sites (forkhead domain—FKHD) region, following the amplification protocol described by SimpleChIP^®^ Enzymatic Chromation IP manufacturer’s Kit. *PLK1* amplification was performed as a positive control for FOXM1 binding. The sequences of the oligonucleotides used were *UBE2C* forward: 5′ CATTGGCTGGATCAAACCCA 3′, *UBE2C* Reverse: 5′ GGAGAACACGACTGCAACTG 3′; *PLK1* forward: 5′ GGGCGGGTTTGGATTTTA 3′, *PLK1* reverse: 5′ AGTCACTGCAGCACTCATGC 3′.

### 2.6. *UBE2C* Promoter Cloning and Dual-Luciferase Assay

The sequence 5′ of *UBE2C* coding regions was analyzed to search for potential FOXM1 consensus regions (FKHD) using JASPAR2016 web-based software [13] and four FKHD were identified. Two of the four FKDH comprised within *UBE2C* promoter were cloned into the promoter-less luciferase plasmid pGL3-Basic (Promega) *Kpn*I–*Xho*I cloning site, generating a *UBE2C*-luciferase reporter system (pGL3-*UBE2C*). The sequences of the primers used to amplify *UBE2C* promoter are: Forward: 5′ AATTGGTACCTGTTCCCACGCGGAGTAAG 3′, Reverse: 5′-AATTCTCGAGGGGGGTGGTCCTAGAAATC 3′. Briefly, TE-1 cells (2 × 10^5^ cells/plate/2 mL) were co-transfected in 6-well plates with different amounts of *FOXM1* expression vector (pcDNA3-FOXM1) and a constant amount of pGL3-*UBE2C* promoter luciferase reporter vector, together with the Renilla luciferase plasmid, using siPORT neoFX Transfection Agent (Thermo). The pRL-TK control vector expressing Renilla luciferase (Promega) was used for normalization of cell number and transfection efficiency. Luciferase activity was measured 48 h after transfection using the dual-luciferase reporter assay system (Promega) with a Lumat LB 9507 apparatus (Berthold Technologies, Bad Wildbad, Germany).

### 2.7. Protein Extraction and Western Blot

Cell protein extraction was performed by washing them twice in ice-cold PBS and subsequently lysing them by using RIPA-like buffer (250 mM NaCl, 50 mM TRIS-HCl, pH 7.4, 0.1% SDS, 2 mM DTT and 0.5% NP-40, (Sigma, St. Louis, MO, USA)) containing protease inhibitors (Roche, Belmonte, CA, USA). Protein concentration was determined by the Bradford assay (Bio-Rad, Hercules, CA, USA) and using bovine serum albumin was employed as standard. An amount of 50 µg of total protein extract was resolved onto a 8.0% SDS PAGE, transferred a nitrocellulose-membrane (Roche) and probed with the appropriate antibodies for 1 h. Antibodies anti-FOXM-1 (Santa Cruz), anti-UBE2C (Boston Biochem, Cambridge, MA, USA) and anti-tubulin (Sigma, St. Louis, MO, USA) were used at 1:250, 1:500 and 1:1000 dilutions, respectively. Next, membranes were incubated with the horseradish peroxidase-conjugated secondary antibody (1:10,000) for 1 h and detection was performed with enhanced chemiluminescence ECL Kit (Amersham, Piscataway, NJ, USA).

### 2.8. In Silico Analysis

In order to analyze *FOXM1* and *UBE2C* gene expression pattern in human healthy tissues, data from sequence read archive (SRA) bioproject PRJEB4337 were downloaded. To investigate *FOXM1* and *UBE2C* expression in a larger set of samples and from distinct tumor types, we accessed and re-analyzed data deposited in the repository The cBioPortal for Cancer Genomics, which responds as an open-access downloaded bio-database, providing visualization and analyzing tools for large-scale cancer genomics datasets. This portal collected records from 147 individual cancer studies, comprising 31 distinct cancer types and over 21,000 tumor samples [14,15]. *FOXM1* and *UBE2C* gene expression data re-analyzed were downloaded from The Cancer Genome Atlas (TCGA) provisional data of 25 different tumor types listed in Table 1. FOXM1 protein levels used in the re-analysis were obtained from the same database. For chromatin immunopreciptation (ChIP)-seq analysis, data of chromatin immunopreciptation followed by high-throughput sequencing were obtained from Encyclopedia of DNA Elements—ENCODE [16], which uses hg19 as the reference genome. Sequences derived from FOXM1 Chip-seq in three human cancer-derived cell lines, MCF-7 (breast cancer), ECC1 (endometrial adenocarcinoma) and SKSH (neuroblastoma) were downloaded and evaluated. The accession numbers of the above cited sequences are wgEncodeEH003288, wgEncodeEH003279 and wgEncodeEH003243 for ECC1, MCF-7 and SKSH cell lines, respectively. For this propose, MACS peak calling [17] was applied aiming to identify the enriched regions of high read density relative to total input chromatin control reads. *PLK1* and *KRT1 loci* were analyzed as positive and negative control for FOXM1 binding, respectively.

### 2.9. Statistical Analyses

To evaluate differences in gene expression between ESCC tissue and their paired nonmalignant surrounding mucosa, a paired *t* test was used when data showed Gaussian distribution and Wilcoxon matched pair test when data did not show Gaussian distribution. To assess the association between gene expression levels, Spearman’s rank for non-Gaussian distribution was employed. All analyses were performed in GraphPad Prism 5 software version 5.02.

## 3. Results

### 3.1. FOXM1 and UBE2C Are Co-Expressed in Healthy Tissues

**Figure 1:**
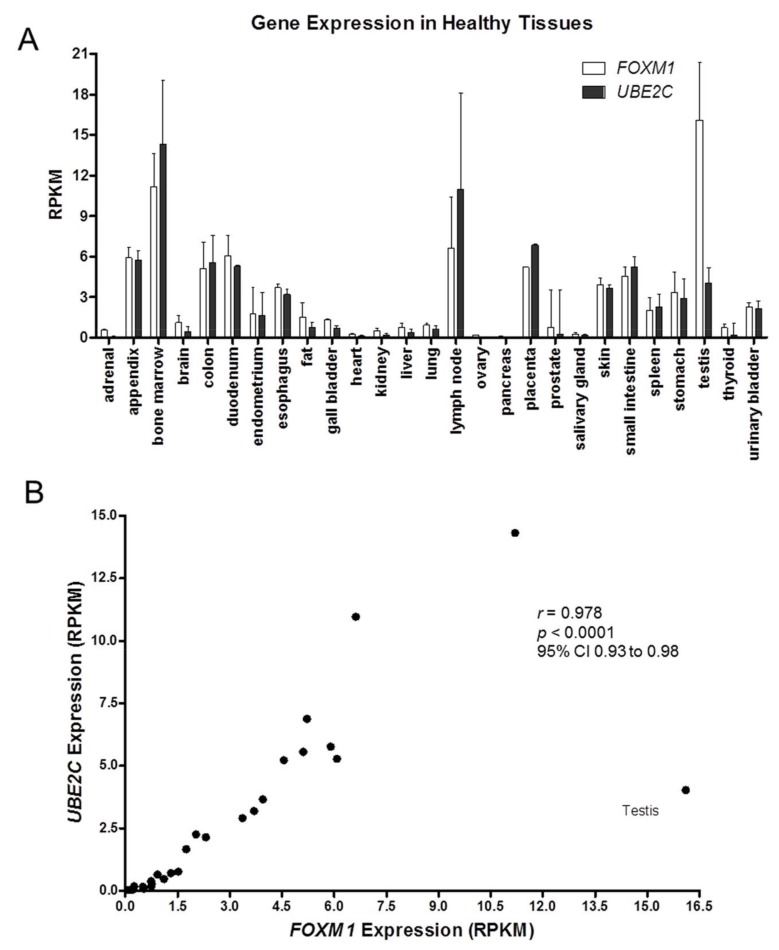
*FOXM1* and *UBE2C* gene expressions are correlated in human healthy tissues: (**A**) *FOXM1* and *UBE2C* show a similar expression profile in several distinct human healthy tissues; (**B**) Correlation analysis between *FOXM1* and *UBE2C* expressions was performed using gene expression data of samples from 27 human healthy tissues using SRA data, bioproject PRJEB4337. Only testis showed a discrepant expression profile. *p* value < 0.05; *r*: Spearman’s rank correlation coefficient; *p*: Spearman’s rank correlation *p* value; CI: confidence interval.

*FOXM1* and *UBE2C* gene expression profiles were analyzed in samples from 27 different healthy tissues, using SRA data, bioproject PRJEB4337, and a very similar expression pattern was observed for these two genes (Figure 1A). In addition, a significant positive correlation (*r* = 0.97; *p* < 0.0001) between *FOXM1* and *UBE2C* median expression values in the different tissues investigated was observed (Figure 1B). 

### 3.2. FOXM1 and UBE2C Co-Overexpression Is a Common Event in Cancer, Including ESCC

**Figure 2:**
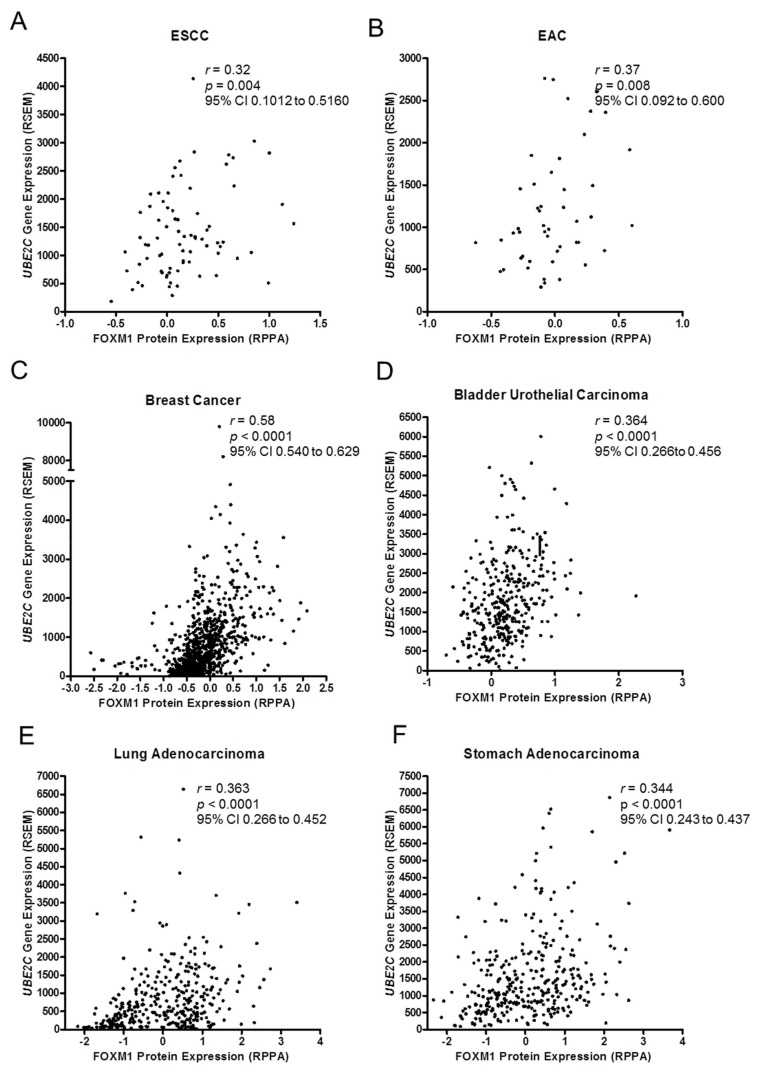
*FOXM1* and *UBE2C* expressions are co-upregulated in cancer. Correlation analysis of FOXM1 protein and *UBE2C* gene expression from TCGA deposited data of different tumor types. A significant positive correlation between FOXM1 and *UBE2C* was observed in 77 ESCC (**A**); 48 esophageal adenocarcinomas (EAC) (**B**); 887 breast cancer samples (**C**); 340 bladder urothelial carcinomas (**D**); 360 lung adenocarcinomas (**E**); and 336 stomach adenocarcinomas (**F**). *r*: Spearman’s rank correlation coefficient; *p*: Spearman’s rank correlation *p* value; CI: confidence interval; RPPA: Reverse phase protein array units; RSEM: RNA-Seq by Expectation Maximization unit.

Next, we re-analyzed *FOXM1* and *UBE2C* expression data from over 7400 samples from 25 different tumor types deposited in the TCGA database and detected a positive correlation between the expressions of the two genes in all tumor types analyzed (Table 1). Furthermore, we also found a statistically significant positive correlation between FOXM1 protein expression levels and *UBE2C* gene expression in some selected tumors: 77 ESCC (*r* = 0.32, *p* = 0.004), 48 esophageal adenocarcinomas (*r* = 0.37, *p* = 0.008), 887 breast cancer samples (*r* = 0.58, *p* < 0.0001), 340 bladder urothelial carcinomas (*r* = 0.36, *p* < 0.0001), 360 lung adenocarcinomas (*r* = 0.36, *p* < 0.0001), and 336 stomach adenocarcinomas (*r* = 0.34, *p* < 0.0001) (Figure 2A–F), suggesting a potential regulation of *UBE2C* by the transcriptional factor FOXM1.

**Figure 3:**
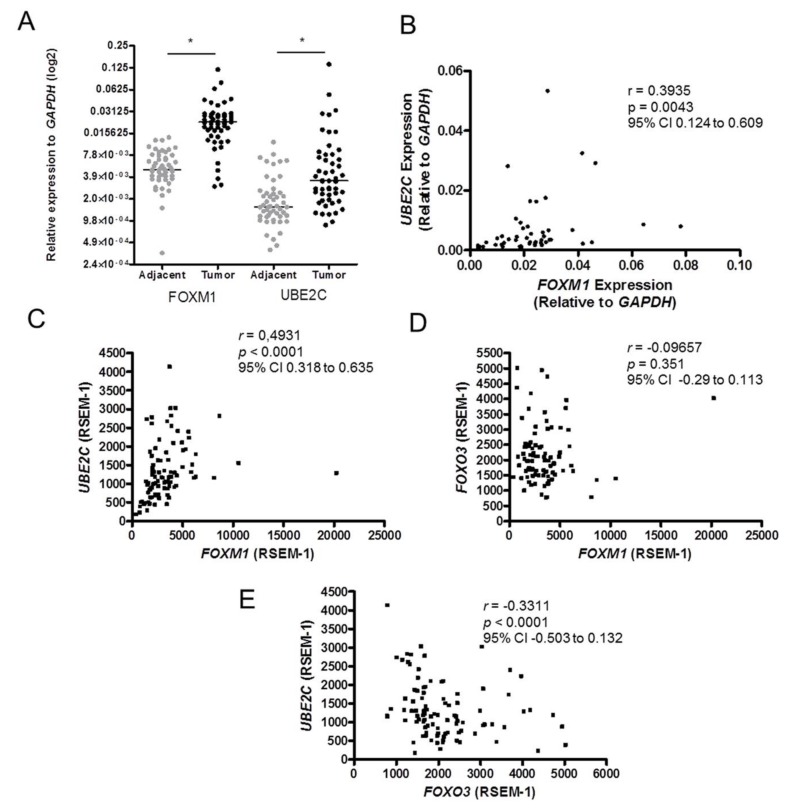
*FOXM1* and *UBE2C* expressions are upregulated in ESCC. (**A**) *FOXM1* and *UBE2C* expression profile in 52 paired ESCC samples, assessed by RT-qPCR; (**B**) Positive correlation observed between the expression of these two genes in the tumor samples analyzed; (**C**–**E**) Correlation analysis of *FOXM1, UBE2C* and *FOXO3* (a functional antagonist of FOXM1) expressions was performed using gene expression data of 95 esophageal squamous cell carcinoma (ESCC) samples deposited into TCGA provisional database; (**C**) Positive correlation between *FOXM1* and *UBE2C* expressions; (**D**) Lack of correlation between *FOXM1* and *FOXO3* expressions; (**E**) Inverse correlation between *UBE2C* and *FOXO3* expressions. * *p* value < 0.05; *r*: Spearman’s rank correlation coefficient; *p*: Spearman’s rank correlation *p* value; CI: confidence interval.

Further, *FOXM1* and *UBE2C* expression profiles were also assessed in 52 paired ESCC and non-malignant adjacent mucosa specimens, showing that both genes were overexpressed (4.5- and 2.2-fold higher, respectively) in tumors, in respect to their non-malignant adjacent mucosa (Figure 3A), and that their expression was positively correlated (*r* = 0.39, *p* = 0.0043) (Figure 3B). This finding was confirmed by the reanalysis of *FOXM1* and *UBE2C* expression data from 95 ESCC samples available in TCGA database (Figure 3C). Moreover, in the same set of samples, the mRNA expression of *FOXO3*, a well-known FOXM1 antagonist [18,19], was not correlated to that of *FOXM1* (*p* = 0.35) (Figure 3D) and inversely correlated to *UBE2C* expression (*r* = −0.33 and *p* < 0.0001) (Figure 3E).

### 3.3. *UBE2C* Figures as a Potential *FOXM1* Transcriptional Target

**Figure 4:**
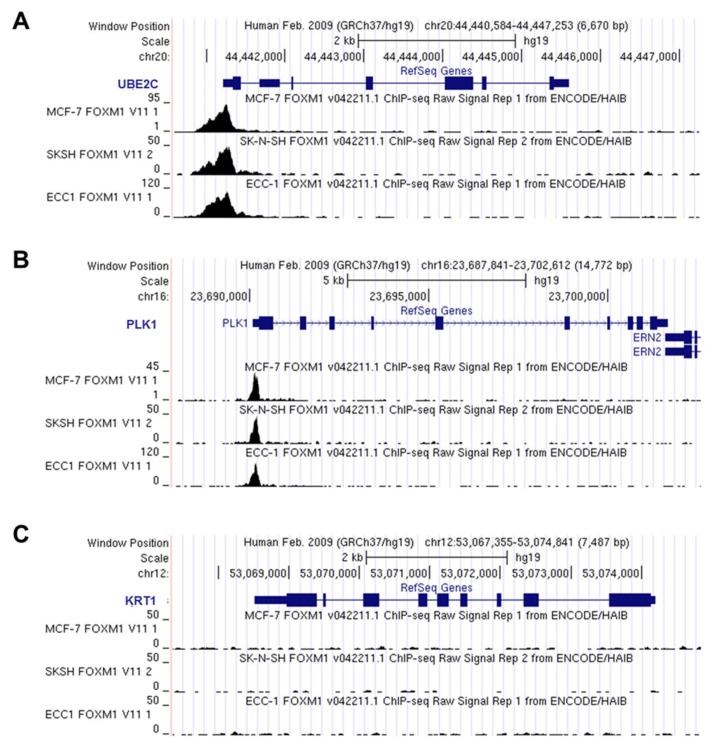
FOXM1 binds to *UBE2C* promoter region. Re-analysis of FOXM1 ChIP-seq data available in ENCODE-USCC platform. FOXM1 chromatin immunoprecipitation was performed in three different human cancer-derived cell lines: MCF-7 (breast cancer derived), ECC1 (endometrial adenocarcinoma derived) and SKSH (neuroblastoma derived), and the presence of DNA peaks were evaluated in *UBE2C* (**A**); *PLK1* (**B**) and *KRT1* (**C**) promoter regions; (**A**) DNA peaks were detected in the region corresponding to *UBE2C* promoter, following FOXM1 immunoprecipitation, in the three cell lines investigated; (**B**) DNA peaks were also detected in the region corresponding to *PLK1* promoter, evaluated as a positive control for FOXM1 binding; (**C**) Absence of DNA peaks in the region comprising *KRT1* promoter, evaluated as a negative control for FOXM1 binding.

Additionally, reanalysis of FOXM1 ChIP-seq data revealed DNA peaks corresponding to *UBE2C* promoter region in three human cell lines: MCF-7 (breast cancer derived), ECC1 (endometrial adenocarcinoma derived) and SKSH (neuroblastoma derived) (Figure 4A), from ENCODE database [16] (accession numbers wgEncodeEH003288, wgEncodeEH003279 and wgEncodeEH003243, respectively). The presence of DNA peaks was also detected in *PLK1* (positive control, Figure 4B), but not in *KRT1* (negative control, Figure 4C) promoter regions, upon FOXM1 immunoprecipitation. These results strongly suggest that *UBE2C* may be a transcriptional target of FOXM1.

### 3.4. FOXM1 Binds onto UBE2C Promoter and Transcriptionally Activates It in an ESCC-Derived Cell Line

**Figure 5:**
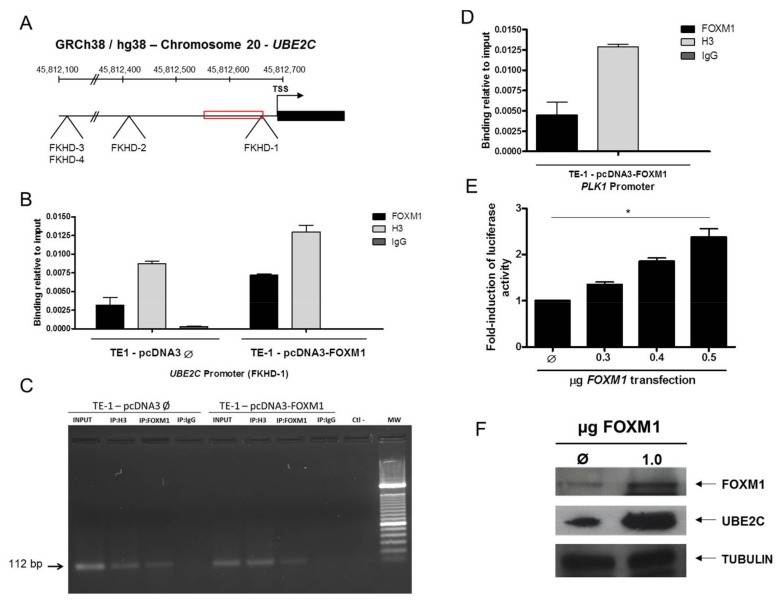
FOXM1 transcriptionally activates *UBE2C* in the ESCC cell line TE-1. (**A**) Schematic representation of the FOXM1 putative response elements position in the 5′ regulatory region of *UBE2C*. Black arrow represents the transcriptional start site (TSS) of *UBE2C.* The putative response elements described are located about −31, −290 and −590 bases upstream of the transcription initiation site and the red frame shows the region encompassed by the primers used to amplify *UBE2C* promoter following FOXM1 immunoprecipitation in TE-1 cell line; (**B**) ChIP assay in the ESCC cell line TE-1 transfect both with *FOXM1* induction expression vector (pcDNA3-FOXM1) and empty vector (pcDNA3). Cross-linked chromatin was immunoprecipitated with anti-FOXM1 antibody (IP:FOXM1) or with anti-histone H3 antibody (IP:Histone H3), used as a positive control, or with anti-normal rabbit IgG antibody (IP:IgG), used as a negative control. Following FOXM1 immunoprecipitation, purified DNA was analyzed by qPCR, using specific primers for −31 to −153 bases of *UBE2C* promoter region encompassing FOXM1 putative response element (FKHD-1). The amount of immunoprecipitated DNA in each sample is represented as a signal relative to the total amount of input DNA. Values are shown as triplicate of the qPCR experiment ± SD; (**C**) In addition, a representative gel image of ChIP results is presented. Ctrl-: PCR negative control; MW—molecular weight (lower marker 100 bases pairs); Black arrow indicates the expected amplified fragment (112 base pairs); (**D**) FOXM1 binding onto *PLK1* promoter was used as positive binding control. The amount of immunoprecipitated DNA in each sample is represented as a signal relative to the total amount of input DNA. Values are shown as triplicate of the qPCR experiment ± SD (**E**) TE-1 cells were co-transfected with *UBE2C*-luciferase reporter (comprising FKHD1 and FKHD2) and increasing amounts of either *FOXM1* expression vector (pcDNA3—FOXM1) or the control (empty) vector pcDNA3 (Ø) and the luciferase assay was performed; (**F**) The ESCC cell line TE-1 was transfected with 1.0 μg of DNA of a *FOXM1* expression vector (pcDNA3—FOXM1) or with an empty vector (pCDNA3-empty), used as control (Ø) and, then, FOXM1 and UBE2C protein levels were analyzed by Western blotting, using tubulin as loading control. The dotted lines delineated the bands selected from the western blotting analysis, according to the molecular weight of the protein investigated. * *p* value < 0.05.

In order to confirm whether FOXM1 is capable of regulating *UBE2C* transcription, we searched for potential FOXM1 binding sites within *UBE2C* promoter region. Three different regions containing four potential consensus forkhead domain (FKHD) sites (FOXM1 binding sites), at base positions −31, −290, −557 and −558 from the *UBE2C* transcriptional start site (Figure 5A), were found. Next, we performed a ChIP assay in the ESCC derived cell line, TE-1, after transfection with both *FOXM1* induction expression vector (pcDNA3-FOXM1) and empty control vector (pcDNA3), and it was observed that FOXM1 binds to a described FKHD site (−31 bp) within *UBE2C* promoter in both conditions. In addition, the binding of FOXM1 onto FKHD site (−31) was 2.2-fold higher in TE-1 transfected with pcDNA3-FOXM1 (Figure 2B,C). As a positive of FOXM1 binding to DNA, the amplification of *PLK1* promoter, a well-known FOXM1 transcriptional target [20], was performed (Figure 5D). Further, we derived one fragment containing 2 FKHD (−31 pb and −290 pb) sites from *UBE2C* promoter and cloned it into luciferase report vector. This vector was transfected together with either increasing amounts of *FOXM1* expression, or control (empty) vector in TE-1 cells. Figure 5E shows a significant increase (up to 2.5-fold, when compared to the control) in luciferase expression in TE-1 cells transfected with both the *UBE2C*-luciferase report and *FOXM1* expression vectors. Finally, TE-1 cells transfected with *FOXM1* expression vector resulted in a higher UBE2C protein expression when compared to those transfected with an empty vector (control cells), as shown in Figure 5F.

## 4. Discussion

This study demonstrates that FOXM1 transcriptionally regulates *UBE2C* expression by directly binding onto its promoter region, and that this may be a common event in healthy tissues and human carcinogenesis. 

FOXM1 is a master regulator of cell cycle progression and its deregulation perturbs the timely and coordinated translation through the cell cycle, leading to a loss of checkpoint control in G1/S and G2/M, by disrupting its transcriptional activity of key genes. Specifically, *FOXM1* overexpression results in an increase of mitotic cell population and induces expression of a large set of G2/M genes [20]. UBE2C acts in the G2/M checkpoint control, possessing a fundamental role in the maintenance of genetic stability by regulating the degradation of securin, a protein that impairs the segregation of the genetic material by binding to and inhibiting the enzyme separase [21]. Its upregulation has been reported in several distinct tumor types, being associated with a highly malignant phenotype and poor survival, suggesting its role in cancer progression [22,23,24,25,26].

The overexpression of either *FOXM1* or *UBE2C* has been reported in a wide range of tumors [3,4,21,24]. In this study, we not only confirmed the overexpression of both genes but showed that their expression levels are highly correlated in 25 different tumor types analyzed, including ESCC. Applying the Bonferroni correction method to identify false positive results, the significance of *FOXM1* and *UBE2C* expression correlation is maintained in all tumors analysed, except for lymphoid neoplasm diffuse large B-cell lymphoma. Strengthening this finding, we observed a very similar expression pattern for *FOXM1* and *UBE2C* gene expression in a range of distinct non-cancerous tissues, as well as a positive correlation between them. Moreover, we detected an inverse correlation between *FOXO3* and *UBE2C* expression in ESCC samples. FOXO3 is a well-characterized antagonist of FOXM1 [18,19], being capable of repressing *FOXM1* expression and also its transcriptional factor activity by preventing the binding of FOXM1 to the DNA [27]. Although there is no correlation between *FOXM1* and *FOXO3* expression, there was a significant inverse correlation between the expression levels of *FOXO3* and *UBE2C,* suggesting that FOXM1 and FOXO3 may be disputing for the binding to the FKHD responsive elements within *UBE2C* promoter region in ESCC.

Re-analysis of data from different cell lines demonstrated that FOXM1 binds to *UBE2C* promoter region, since DNA peaks corresponding to this region were detected. Subsequently, in vitro assays conducted in an ESCC-derived cell line clearly showed that FOXM1 binds onto *UBE2C* promoter region and that FOXM1 induced expression resulted in both increased luciferase activity driven by *UBE2C*-promoter and increased levels of UBE2C protein. Together, these results demonstrate that FOXM1 transcriptionally regulates *UBE2C* expression. Corroborating our results, a very recent study reported that FOXM1 binds onto *UBE2C* promoter and triggers its transcription in glioma cells and, therefore, protects them from autophagic cell death [28]. It is worth mentioning that, in our TCGA gene expression data re-analysis, the highest correlation coefficient observed between FOXM1 and UBE2C gene expression was in low grade gliomas, and the above cited study [28] supports our hypothesis that the co-overexpression of both genes and the transcriptional regulation of UBE2C by FOXM1 may a general phenomenon in tumors.

Although we have observed promoter activation and a consequent increase in UBE2C gene and protein levels upon *FOXM1* overexpression in TE-1 cells, its silencing was not capable of diminishing *UBE2C* expression levels. The same was observed for *PLK1* gene [20]. Similarly, re-analysis of gene expression microarray data deposited in Gene Expression Omnibus Database (GEO-NCBI) from three independent studies using breast cancer cell lines (MCF7—accession number GSE55204, MDA-MB-231—accession number GSE25741 and BT-20—accession number GSE2222) revealed that *FOXM1* silencing was not capable of altering both *UBE2C* and *PLK1* gene expression (Supplementary Materials Figure S1). Taken together, these results suggest that other transcriptional factors and/or mechanisms may play a role in *UBE2C* transcriptional regulation. In this sense, analyses conducted using The Encyclopedia of DNA Elements Consortium [16] data indicated that 54 different transcription factors were detected bound to *UBE2C* promoter in a wide range of different cell lines, supporting the hypothesis that several proteins may participate in *UBE2C* expression control, both in solid and liquid tumors. Thus, for instance, FOXA1 (Forkhead box protein A1), another member of the forkhead box protein family, was found bound in the exon 3 region of *UBE2C* gene and, in association with MED1 (mediator complex subunit 1), was capable of altering *UBE2C* expression levels in prostate cancer cells [29]. Furthermore, *E2F4* (E2F transcription factor 4) overexpression resulted in lower levels of *UBE2C* expression and in a decrease of its promoter activity in HCT116 cells [30]. In addition, in a leukemia model in which myelodysplastic syndrome mice were used, *MYBL2* (MYB proto-oncogene like 2) knockdown also resulted in decreased *UBE2C* expression [31]. Thus, although in our study we have demonstrated that FOXM1 transcriptionally regulates *UBE2C*, our new data, as well as data from the literature suggest that it might not be the only mechanism by which *UBE2C* expression is controlled. In this way, the impact of *FOXM1* silencing on *UBE2C* expression could be offset by other transcription factors and other proteins associated to its gene expression control. 

Further, other molecular mechanisms have already been accounted for regarding *UBE2C* overexpression. For instance, amplification of the 20q13.1 locus has been reported as one of the main mechanisms related with *UBE2C* aberrant expression, being observed in several malignant tissues, including colon, thyroid, prostate, nasopharynx and esophagus [26,27,28,29,30,31,32,33,34,35]. Of note, these tumors also show *FOXM1* overexpression [36,37,38,39] and exhibited *FOXM1* and *UBE2C* expression correlation in the analyses herein presented, indicating that FOXM1 may play a role in *UBE2C* expression regulation together with 20q13.1 *locus* amplification. Moreover, the expression of both *FOXM1* and *UBE2C* in breast cancer has already been associated with *ErbB2* pathway. HER2 signaling cascade increases *FOXM1* expression [40] whereas *ErbB2* silencing leads to decreased levels of *UBE2C* [41]. Interestingly, once more it is possible to observe the involvement of FOXM1 in another described regulatory mechanism of *UBE2C* expression. Additionally, it is worth mentioning that our data pointed out a positive correlation between the expression of both genes, as well as of FOXM1 protein and *UBE2C* gene expression, and also demonstrated that FOXM1 binds onto *UBE2C* promoter in a breast cancer cell line. In this way, it is feasible to state that FOXM1 is a crucial regulator of *UBE2C* expression and may play a role even in the presence of other regulatory mechanisms. 

It has been reported that wild-type p53 mediates the repression of *UBE2C* and *FOXM1* expressions, while its mutant form acts in the opposite way [30,42]. *TP53* mutations that result in loss of function of p53 are the most frequent and homogenously detected molecular alterations in ESCC [43,44], occurring in early stages of ESCC carcinogenesis [45]. Consequently, lack of p53 activity may lead to an increase of FOXM1 protein expression and, consequently, of its targets, such as *UBE2C*, contributing to the loss of proper cell cycle control, a phenomenon highly present in ESCC [44]. In fact, we have previously shown that *UBE2C* silencing in two ESCC-derived cell lines leads to a decrease in cell proliferation rates and alters cell cycle profile, by interfering with cyclin B1 levels [10].

## 5. Conclusions

In this study, we demonstrate that *UBE2C* is a transcriptional target of FOXM1 in ESCC, and likely present in several human neoplasias, thus further contributing to the loss of G2/M checkpoint control as a consequence of FOXM1 deregulation.

## Figures and Tables

**Table 1:** Correlation between forkhead box protein M1(*FOXM1*) and ubiquitin conjugating enzyme E2 (*UBE2C*) gene expression in several different human tumors.

Tumor	Number of Samples	Spearman *r*	*p* Value
Acute myeloid Leukemia	197	0.7	<0.0001
Adrenocortical carcinoma	92	0.87	<0.0001
Bladder urothelial carcinoma	413	0.63	<0.0001
Brain low grade glioma	530	0.94	<0.0001
Breast cancer invasive	1100	0.85	<0.0001
Cholangiocarcinoma	35	0.79	<0.0001
Colorectal adenocarcinoma	382	0.47	<0.0001
Glioblastoma multiforme	166	0.71	<0.0001
Hepatocellular carcinoma	373	0.82	<0.0001
Head and Neck Squamous cell carcinoma	522	0.46	<0.0001
Kidney renal clear cell carcinoma	534	0.84	<0.0001
Lung adenocarcinoma	517	0.78	<0.0001
Lung squamous cell carcinoma	177	0.38	<0.0001
Lymphoid neoplasm diffuse large B-cell lymphoma	28	0.29 *	0.0389 *
Mesothelioma	86	0.83	<0.0001
Ovarian serous cystadenocarcinoma	307	0.58	<0.0001
Pancreatic adenocarcinoma	179	0.81	<0.0001
Pheochromocytoma and paraganglioma	184	0.8	<0.0001
Sarcoma	262	0.77	<0.0001
Skin cutaneous melanoma	471	0.74	<0.0001
Stomach adenocarcinoma	415	0.6	<0.0001
Testicular germ cell cancer	156	0.62	<0.0001
Thymoma	120	0.77	<0.0001
Thyroid carcinoma	509	0.79	<0.0001
Uterine corpus endometrial carcinoma	177	0.52	<0.0001
Uveal melanoma	80	0.9	<0.0001

Results generated by analyzing expression data deposited into TCGA database. r—Correlation coefficient; * Pearson correlation analysis.

## Supplementary Materials

The following are available online at http://www.mdpi.com/2073-4425/9/4/188/s1, Table S1: Clinicopathological characteristics of the 52 esophageal squamous cell carcinoma (ESCC) patients comprised in this study; Figure S1: The impact of *FOXM1* silencing on gene expression levels of known transcriptional targets; Supplementary ChIP and luciferase assays data.
