# Supplementary material for: UBE2C Is a Transcriptional Target of the Cell Cycle Regulator FOXM1
Source: Genes (Basel). 2018 Mar 29;9(4):188. doi: 10.3390/genes9040188 (PMC5924530; doi:10.3390/genes9040188)
Supplement: Supplementary file 1 [file genes-09-00188-s001.zip › Figure S1.docx]

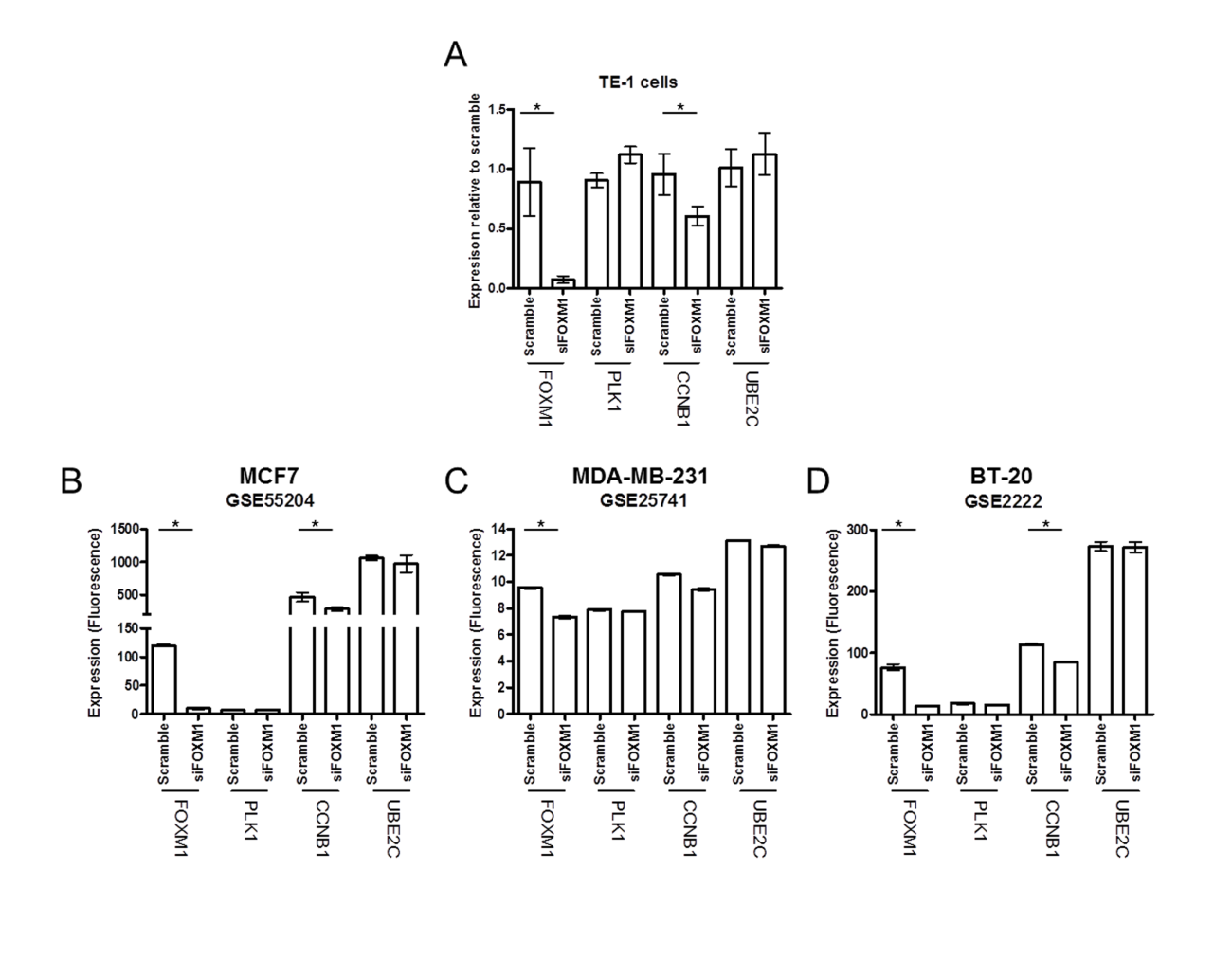


Figure S1: The impact of *FOXM1* silencing on gene expression levels of known transcriptional targets. *FOXM1* silencing was not capable of diminishing *UBE2C* and *PLK1* expression in TE-1 cells. However, *CCNB1* expression was reduced upon *FOXM1* silencing (A); Re-analysis of gene expression microarray data deposited in Expression Omnibus Database using MCF7 (B); MDA-MB-231 (C) and BT-20 (D) cell lines revealed similar results to those observed in TE-1 cells. * *p* value < 0.05
