# Supplementary material for: UBE2C Is a Transcriptional Target of the Cell Cycle Regulator FOXM1
Source: Genes (Basel). 2018 Mar 29;9(4):188. doi: 10.3390/genes9040188 (PMC5924530; doi:10.3390/genes9040188)
Supplement: Supplementary file 1 [file genes-09-00188-s001.zip › Supplementary ChIP and luciferase assays data.docx]

Supplementary ChIP and luciferase assays data

The active promoter region of *UBE2C* gene was characterized in the UCSC Genome Browser, using the Human Genome GRCh37/hg19 and data from Chromatin State Segmentation by HMM from ENCODE/Broad. The FASTA sequence was downloaded and analyzed in the web-based software JASPAR 2016 aiming to identify response elements to forkhead proteins in the *UBE2C* promoter.

FASTA Sequence: >NC_000020.11:44436334-44442334 Homo sapiens chromosome 20, GRCh37/hg19 Primary Assembly

CCAGCACTTTGGGAGCCCGAGGCAGGCGGGTCACCTGAGGTCAGGAGTTCAAGGCCAGCCTGGCCAACAT

GGTGAAACCCCATCTCTACTAAAAGTACAAAAATTAGCCGGGCATGGTGGTGCACTCCTGTAGTCCTAGC

TACTCTACTCGGGAGGCTGAGGCAGGAGAATTGCCTAAACCCAGGAGGCAGAGGTTTCAGTGAGCCGAGA

TCACGCCACTGCACTCCAACCTGGGCGACAGAGCAATACTCTGTCTCAAAAAATATATATATCAGTTTCT

CTTCACTCCTCGATTTTTTTTTTAAGGTGCTTAAATTCTGGTTTCTAGGTGCCCAACCTAGAATAGAAAC

CAGAATTTAAGCCTTATTTGGGTTGAGCACAGTAGCTCATACCTGTAATCCCAACACTTTGGGAGACTGA

GTGGGTGGATCAGTTGAGGCCAGGAGTTCAAAACCAGCCTGGCCAACATGGCAAAACCCCATCTCTACTA

AAAATACAAAAATCAGCTGGGTGTGTTGGCACGTGCCTGTAATCCCAGCTATTCAAGGCTGAAGCAGAAG

AATAGCTTGAACCCAGGAGGTGGAGGTTGCGGTGAGCCAAGATCATGCCACTGCACTCTAGTCTGAACAA

CAGAGCGAGACGTCATCTCAATAAATAAATAAACAGCCAGGCACGGTGGCTCACACCTGTAATCCCAGCA

CTTTGGGAGCCCGAGGTGGGCAGATCACCTGAGGTCGGGAGTTCAAGACCAGCCTGACCAACATGGAGAA

ATCCCATCTCTACTAAAAATACAAAATTAGCCGGGCGTGGTGGCGCATGCCTGTAGTGCCAGCTACTTGG

GAGGCTGAGGCAGGAGAATCGCTTGAACCCAGGAGGCGGAGGTTGTGATGAAGCAAGATCATGCCATTGC

ACTCCAGCCTGGGCAACAAGAGTGAAACTCCGTCTCAATCGATCAATCAATCAATCAATCAATAAACCAA

CCAACCTTATTTGTAACTCCAAAACCGAATTCTTAACCATGATGCTATTATGCGTCCGAGAGTACATGAG

GTGTCCCGATTTTTGTTTTATTATGGTCACCATAATAATTACAGACGTTATAAGCCACCTCCGATCATTT

TTTAAAAAGAGGAAGCTCTAAAATAATAGTTATCAGTCCCACCAGTTGCCCGGGGTGCTTTGTTGTGAGA

AGGGGGTGGGGATTGGGAGTATACTAAGTCTCTGGGAATTGGGTCCCATGGTCTGCCCTCGGGGTTGGCT

TAGCTTTCTCTGAAGAGATTAGTGAGCCCTCTAAGTCCACCACGCTTGGAGACAAGGACTTTTGGTAAGA

ACTGCTCAAGGGCCAAGAGTATGCTCTGGGACCAAATGAGAAAAGCCCCTTACCCGAGGCTAATTTTCTT

ACAACTTCATTCCTTACAGTATGCAGCTGACCCCCAGGATAAGCACTGGCTGGCTGAGCAGCATCACATG

CGGGCAACAGGGGGCAAGATGGTAAGCATTATTCATTTGTGCCACTGCCAGTGACCCACCCCACCTGGGA

ACCAGGATTTTGGCTGTGGGTGACAGCCTACCTCCAAATCTGACTTCCAAAGCCTCACCAATGTGTGAGA

AGAGAGACTTATAAGGCCTATTTCTTCATGCTGAAGAGCAAATTGTAATCTTCAGGTTCCCTTCCCTATC

CCTAGGTCTCCCTTCCGCCCCCTCACACTTTTACCTCATGCCTCCAAGATCACATATTTGGATACTATTA

GCTGTCCATAGGAATAGCAGGTATAAGACACACAGTACTACTTTTTCCCACTACTGCACCCACTGACAGA

TTTCACTCATTGATCACTCTTCCACCAGACACAGCTATAGAATCTTTTCTCAACACAGTACTTGAGGCAG

CAACCCTTGTCAGTAGGAGTTAGCAATGAAATGTGTTTGCACACTTGGCAGCCCCTTCCAGATTTACCTC

TACTTGAAGAGTTCTCAGATCATATGAAACAGCAACCTTAGGAAAATCATCATTGCATGTGTTCAAAAAC

TATTGTTGAGGGCTTTGTCCATCACACACAATGTCCCCCACCTGAGGACTCACTCACTCCCACCACAGGT

ACTGCTCCTTTTAGAATCTAAGTGCCTTACAAACTGATATGCCATCTCCTTTCTGGCAGTGAGAGCTACC

TTTTATTTTATTTTACTACTCTATTTATGCCTATGCTTTACTTCAAAAAAGATTTCAGGTAAGCTTCATG

CACATAAAATGCAACATTACATAAATAAGAAATAGATGGTAGGGGGTGGGGGCAGGACCTAAAATTAAGC

CACCAGTAAAAGTAGTTCTTTAAAAAAAAAAAGTGTTTTTTATATTGCTGACCTGGGAGACCTGTGCACT

TCTACAGATGTTGGCAAATTCAGCTGTAGGCCCCACTCATGGACACCTGATCTGTTATGGTTCATAGTGC

CCATAAGATGTAGACAAATTACTCAAGGAATACACAATTATTCCTGACCCTAACATTAGAAAGTCTCCCA

CAGAAGACTTTCTAATATGATGAATATCTCTTCCTATATGTTAAGTTCACAAAAGGGCCCTCACCCACTG

TAGACCGAGGCATTGTGACAAAGCATTTCTACAGGCCCCAGTACAATACAGGCCAATTACTAGACAGCAG

AGCAGTTTATCATGTTTTCCATTTTGCCACAAAGTTCAAAACCAAGTTATCTTTGTCCTCAGAGGGAGCA

CATGGTTTCCTCTTCCTGATTTCCTGTTTCCATATATATTTTGCTGGCTTTCTTGCCATTTTGTTATAAC

CCACCTTGGGTCCTTTCGTATACTTGATCTTAACCAAAAGGTGTAGGAGCAATGGTGGATCCCTTTTTTT

TTCTTTTTTTCTTTTTTTTTTTTTTTTCTTAATTTTCACCACACCATTCAAGAGAGAAATGGGTCCTTTT

TTAAAAGAGAAGGCTATAAATAAGTCACCAGTCCCTACTCTCCCATCCATGAAGCATCTACTGTAGTAAA

AGATTGTCTCAAGGAACCATTCCCATCCCTCCCCACTTACTCTTCCGTACTAAATTGTAAACCGCATCCC

CCGCTTCCACCTAGTAGCAGGCCTTACATTAAACAGATGTTTAATGAAGTGTTACTGAGTTGGAGTGAAA

TGAATCAGGCAATGACCACTCTCCCTTGCTCCTGCCTCAGGCCTACCTCCTCATCGAGGAGGACATCCGG

GACCTTGCGGCCAGTGATGATTACAGGTAAAACCAGTGGGTCTCCTGCCCCTTCTCCTCTCCCTGCCTCC

AAATAGCCCCTAGAATGAGGCAGGGCCTACATCCTCACTTCCCCCAACTTCTCTCTTCAATGTCTGTTCC

AGAGGATGCCTGGATCTGAAGCTAGAGGAATTGAAATCCTTTGTCCTACCCTCCTGGATGGTGGAGAAGA

TGAGAAAGTATATGGAGACACTACGGACAGAGAATGAGCATCGTGCTGTTGAAGCACCTCCACAGACCTG

AGGCCGGGTCCCCTGGCCACACTTGGCAGCCCTCCTCCAAAGCCCTCTTCCTCACGTGGCTGAGGCCACC

GCTGGGACTGCTCCTAGATGGATCTCAGCGGCATTAAGCTGTGCCTGAGCGAGTTTGTAGTGACTCACTG

CACAGCACCCCCAGACTAGCATGTGGTTCTATATTTGTAAAGTTATTGGGATAAGAAACAATTAAACAGT

TTGTAGTAAACACAGATGGTGAACCTGCTGTGCCCTCTACCTTGTGGGAATTGACAGAACATCAAGGGCT

CTAGAAGTGGGTGTAGGAAAAAAGGACGAGATAACCCTCACCCATAACAGTATAGAGCCAGGCTTGATAA

GACCAACCTGGGAGCACCATGTACCCTGCCCGTCTTCCCTTTGCCCATTTGTAGTTTCCTTACCCAGCTA

ATGTAAGGACATGGCAGAATGCGATGGAGTTTGATGACAGATCATATGAAAAATAGAGTTGGCTTTTATT

GCTCCCATTTTACAGGTTAAGATACTAAAGGACCCGCCCCAAGGTTACACACTACCCACTGGGGAGGTAG

GATATACAGGATATGAACCTGTGTTGTTGGGGTTTTTGTTTGTTTGAAGACAGTCTTGCTCTGTTGCCCA

GGCTAGAGTGCAGCCTCTGGCTCAAGCGATTCTCCCGCCTCAGCCTCGCCAGTAGCTGGGACTACAGGCT

CGTGCCACCTCACCAGCCTTTTTTTTTTTTCTTTTCTTTTTTTTTTTTTTTCTTTTAAGTAGAGACAGGT

CTCCCTATCCTGTTGCTCAGGCTGGTCTCGAACTCCTGGGTTCAAGTGATCCTCCTGCCTGGGCCTTCCA

AAGTGTTCGGGATTACGGACGTGAGCCACTGCGACCAGCCTGAACCAATGTTGCCTTTTTTTATTTCTGT

CATCTCTGCGGAGATGTATGGACCTATGTTTTCTAATAGGGAGCCTGCTGCTCTGGCCATCAGCCTTTAG

TTTTTCCCCTCGCTGGGATTTTCAGATACTTAGGACGCAGCAAAGGTGCCCAGGCGATTATAATGGTGGG

AGGGTACCAGTGCTGCAGGATTGGAGGCACTCCCTGCCCGAGGGAAATTGGATCAAGGCTGGGCGTGCCG

AGTGTTCCCACGCGGAGTAAGACGTGTAGCCAGAAGGAGGGGTTCATCCCACGTGGACGTTTTCTTGGCC

CTTTAATGGTTAGCGTTTTCCAACGGCCACCAATTCGCTACGGATTCGTTAGTTAATGGCAGCATCATCT

ACCAATCGGTTGTCAGAAGCGGGATTCTGCCGCCCAATAGCGGTGCGCCAGTGGGTAGGTCTAGCAGTGG

CGCAGCAATAGAGCGCTCCGGAGCGTCTCATTGGCTGGATCAAACCCAAGCGAGCCATTGATTGGTCGAC

GCCCCCAGAGGGTTACAATTCAAACGCGGGCGGGCGGGCCCGCAGTCCTGCAGTTGCAGTCGTGTTCTCC

GAGTTCCTGTCTCTCTGCCAACGCCGCCCGGATGGCTTCCCAAAACCGCGACCCAGCCGCCACTAGCGTC

GCCGCCGCCCGTAAAGGAGCTGAGCCGAGCGGGGGCGCCGCCCGGGGTCCGGTGGGCAAAAGGTGAGTGA

TGCGGCCTACCACTCGCCGGGCCTGCCATGCCCTAGGCATTGGTACCCAGAGCAAAGATTTCTAGGACCA

CCCCCCGCCGCCACCTCCTGGAGCGGGAGATCTGCGGGTGCAGGAGAACACACCAGGAGCTCGGGGCCTG

GCATTCCCCTGGGCATGGGTGTCGAGGGCGGAGACTTCCGGGACTCCCACCTCCTTGCGCGGGTGAGATT

CGAGAGATGGGGGACAGGCCAGGAGCTCAGACCGCTCTTTGAGACTCTCCCGAAGGAGAATGGGAGGGTA

GGGGCGCTGCCAGACTCCTTCCCTGGTGGGCCTAGATGAAGACGCTCAAGGACCCTCGTGACTTGGCCGA

GACAGGGGAAGGGAGAAGTTGAGTCGGGCAAGGAAGAGATGCTAAAGCCTGGGGAATTAAGAACATGCCA

GAATCATCCCGAGGGAGTCTGGAATTAGGGAGGGTGAGGACTCGCTAGGATCGTCCTGTGGATCTGGTGA

GTATACCCTGAGCTGAGCCTGACCTTGGTGATGTGGGGAGCCTCCATGACGGCTCCCCCTGAGATTCAGG

TGGGAGAAGATGCACCGTCTGGAGGCCTGGCCCATCCAGACTCCCAGGTAACCCCGAATACTCTTTTTTC

AGGCTACAGCAGGAGCTGATGACCCTCATGGTGAGTGATTAAGTGCCCAGAACCCCAGCCTTCCATCCAA

TTTTCAGTAGCCTCCTTTTTTCCGTCAGCTTTTTTGCTAGACATAGGGGTAATGTAATTTGCTCCCTCCT

GGGAAAGAAGTTCATACACCCCACCTACACCATTTCTTCCAGCAGTCCCTCCTCCCAATTCCATCCCCCC

ACACGAAGTTATCTCGAACACTTCCCTGAAGTCATACAAGACCCTCCCTAT

Transcriptional Start Site position: 44,441,335

The position of the FORKHEAD DOMAIN (FKHD) identified in the *UBE2C* promoter (in reference to the transcriptional start site):

1- -31 (chr20:44,441,304)

2- -290 (chr20:44,441,045)

3- -557 (chr20:44,440,778)

4- -558 (chr20:44,440,777)

Primers used to qPCR of ChIP assay:

*PLK1* – NC_018927.2

Forward primer: GGGCGGGTTTGGATTTTA

Reverse primer: AGTCACTGCAGCACTCATGC

*UBE2C* – NC_000020.11

Forward primer: CATTGGCTGGATCAAACCCA

Reverse primer: GGAGAACACGACTGCAACTG

In the luciferase assay, to clone the *UBE2C* promoter region comprising the FKHD 1 and 2 the following primers were used:

Forward primer: AATTGGTACCTGTTCCCACGCGGAGTAAG

Reverse primer: AATTCTCGAGGGGGGTGGTCCTAGAAATC
