# Supplementary material for: UBE2C Is a Transcriptional Target of the Cell Cycle Regulator FOXM1
Source: Genes (Basel). 2018 Mar 29;9(4):188. doi: 10.3390/genes9040188 (PMC5924530; doi:10.3390/genes9040188)
Supplement: Supplementary file 1 [file genes-09-00188-s001.zip › Table S1.doc]

Supplementary Table 1: Clinicopathological characteristics of the 52 esophageal squamous cell carcinoma (ESCC) patients comprised in this study.

N/A = not informed

| **Clinicopathological Features** | **Frequency** |
| --- | --- |
| **Age** |  |
| Median (years) | 59 (39 - 79) |
| <59 | 25 (48%) |
| ≥59 | 27 (52%) |
| *Total* | *52* |
| **Gender** |  |
| Male | 40 (76.9%) |
| Female | 12 (23.1%) |
| *Total* | *52* |
| **Smoking** |  |
| Former | 9 (17.3%) |
| No smoker | 8 (15.4%) |
| Smoker | 33 (63.5%) |
| N/A | 2 (3.8%) |
| *Total* | *52* |
| **Alcoholism** |  |
| Former | 10 (19.2%) |
| No alcoholic | 8 (15.4%) |
| Alcoholic | 32 (61.5%) |
| N/A | 2 (3.8%) |
| *Total* | *52* |
| **Death** |  |
| No | 16 (30.8%) |
| Yes | 36 (69.2%) |
| *Total* | *52* |
| **Tumor Site** |  |
| Lower Third | 8 (15.4%) |
| Medium Third | 30 (57.7%) |
| Upper Third | 14 (26.9%) |
| *Total* | *52* |
| **Clinical Stage** |  |
| I | 2 (3.8%) |
| II | 15 (28.9%) |
| III | 20 (38.5%) |
| IV | 6 (11.5%) |
| N/A | 9 (17.3%) |
| *Total* | *52* |
| **Tumor Differentiation** |  |
| Low | 13 (25%) |
| Moderately | 37 (71.2%) |
| Well | 2 (3.8%) |
| *Total* | *52* |
